# Trypsin Potentiates Human Fibrocyte Differentiation

**DOI:** 10.1371/journal.pone.0070795

**Published:** 2013-08-07

**Authors:** Michael J. V. White, Melissa Glenn, Richard H. Gomer

**Affiliations:** Department of Biology, Texas A&M University, College Station, Texas, United States of America; University of Pittsburgh, United States of America

## Abstract

Trypsin-containing topical treatments can be used to speed wound healing, although the mechanism of action is unknown. To help form granulation tissue and heal wounds, monocytes leave the circulation, enter the wound tissue, and differentiate into fibroblast-like cells called fibrocytes. We find that 20 to 200 ng/ml trypsin (concentrations similar to those used in wound dressings) potentiates the differentiation of human monocytes to fibrocytes in cell culture. Adding trypsin inhibitors increases the amount of trypsin needed to potentiate fibrocyte differentiation, suggesting that the potentiating effect is dependent on trypsin proteolytic activity. Proteases with other site specificities such as pepsin, endoprotease GluC, and chymotrypsin do not potentiate fibrocyte differentiation. This potentiation requires the presence of albumin in the culture medium, and tryptic fragments of human or bovine albumin also potentiate fibrocyte differentiation. These results suggest that topical trypsin speeds wound healing by generating tryptic fragments of albumin, which in turn potentiate fibrocyte differentiation.

## Introduction

The failure of wounds to heal properly constitutes a major medical problem, with both acute and chronic wounds consuming treatment time and resources. Between 25–40% of hospital patients receive treatment for either an acute or chronic wound [1]–[5]. Poorly-healing chronic wounds affect more than 6.5 million US patients per year, and cost more than $25 billion to treat [6]. Chronic wounds resist conventional wound-dressing treatments and often occur in elderly, obese, immuno-compromised, or diabetic patients [6], [7].

Monocytes are circulating cells that are recruited to wounds and sites of tissue injury by chemokines [8], [9]. To help heal wounds, monocytes enter the wounded tissue and differentiate into fibroblast-like cells called fibrocytes [10], [11]. Fibrocytes are collagen-expressing CD45+ cells which assist in scar tissue formation, a key component of both wound healing and fibrosing diseases [10], [12]–[14] and the development of the body’s desmoplastic response to foreign or invasive bodies [12], [15]. Increasing fibrocyte differentiation within a wound healing environment potentiates wound healing, and decreasing fibrocyte differentiation leads to slower wound healing [16]. Monocytes isolated from peripheral blood mononuclear cells differentiate *in vitro* in a defined media into fibrocytes [17]. The differentiation can be modulated by factors such as the serum protein Serum Amyloid P (SAP), salt concentration, cytokines such as interleukins 4, 12, and 13 and interferon, or hyaluronic acid [18]–[21]. Increased fibrocyte differentiation correlates with increased fibrosis and wound healing in animal models [16], [22].

After wounding, the blood clots, leaving serum on the wound. Albumin is the most common protein in serum, with levels between 35–50 g/L and accounting for ∼50% of the total protein in blood [23]. Albumin is produced in the liver and maintains blood homeostasis [23]. Increased albumin levels are associated with increased wound healing in both acute [24] and chronic wounds [25]–[27].

Trypsin, chymotrypsin, and pepsin are mammalian proteolytic enzymes. Each is secreted as a zymogen, and later cleaved to an active form. Each also cuts at a different amino acid pattern. Trypsin is a proteolytic enzyme that cuts at lysine or arginine residues, except when the following amino acid is proline [28]. Chymotrypsin preferentially cleaves following the aromatic amino acids tyrosine, tryptophan, or phenylalanine [29]. Pepsin cuts preferentially between hydrophobic and aromatic amino acids [30]. Endoproteinase Glu-C is a protease isolated from *Staphylococcus aureus* which cleaves at glutamic or aspartic acid [31], [32].

Although trypsin is generally thought of as a digestive enzyme, trypsin is also active in multiple cellular processes, including development and tumor invasion [33]–[42]. Trypsin is involved in gastric inflammation through cleavage of the proteinase-activated receptors (PAR1–4) [43]–[45], and PAR1 and PAR2 digestion has also been implicated in cancer signaling [46], [47]. Topical application of trypsin has been used to potentiate healing of both conventional and chronic wounds for more than 50 years after having been initially tested as a burn debridement treatment [48]–[54]. In this report, we show that trypsin potentiates fibrocyte differentiation, suggesting a mechanism of action for the effect of trypsin on wound healing.

## Materials and Methods

### Cell Isolation and Exposure of PBMCs to Proteases and Inhibitors

Human blood was collected from adult volunteers who gave written consent and with specific approval from the Texas A&M University human subjects Institutional Review Board. Peripheral blood mononuclear cells (PBMC) and monocytes were isolated as previously described [18], and monocytes were checked for enrichment by flow cytometry in comparison to the un-enriched PBMC population [55]. Cells were cultured in Fibrolife basal media as previously described [17], in either protein-free media (PFM) or serum-free media (SFM). PFM is composed of Fibrolife basal media (Lifeline Cell Technology, Walkersville, MD) supplemented with 10 mM HEPES (Sigma), 1×non-essential amino acids (Sigma), 1 mM sodium pyruvate (Sigma), 2 mM glutamine (Lonza), 100 U/ml penicillin and 100 µg/ml streptomycin (Lonza). SFM is composed of PFM supplemented with 1×ITS-3 (500 µg/ml bovine serum albumin, 10 µg/ml insulin, 5 µg/ml transferrin, 5 ng/ml sodium selenite, 5 µg/ml linoleic acid, and 5 µg/ml oleic acid, (Sigma). Where indicated, PFM was supplemented with recombinant insulin suitable for cell culture (Sigma), transferrin suitable for cell culture (Sigma), or human or bovine albumin to the above concentrations, or 12.5% human serum. TPCK is an irreversible inhibitor of chymotrypsin, and TLCK is an irreversible inhibitor of trypsin. TPCK-treated trypsin (Sigma), TLCK-treated chymotrypsin (Sigma), pepsin from porcine stomach mucosal lining (EMD), or endoproteinase Glu-C from *S. aureus* (Sigma) were all resuspended to 10 mg/ml following the manufacturer’s instructions. Complete protease inhibitor cocktail (Roche, Indianapolis, IN) was resuspended to 40 mg/ml in water, and soybean trypsin inhibitor (Sigma) was resuspended to 10 mg/ml in water. Fibrocytes were stained, identified and counted as previously described [56].

### Purification of Albumin

Albumin was purified from sterile filtered non-blood type specific human serum, tested negative for hepatitis A and B and HIV I and II (Lonza, Basel, Switzerland and Gemini Bio-products, West Sacramento, California) or from triple filtered US origin fetal calf serum, tested for sterility and mycoplasma (Thermo Fisher Scientific, Milwaukee, WI) by affi-gel bead affinity elution (Bio-Rad, Hercules, California). 4 ml of beads were washed three times in 25 ml PBS, and were added to 40 ml of serum with gentle mixing at room temperature for 2 hours. The beads were collected by centrifugation at 300×g for 5 minutes and washed three times with 25 ml of filter-sterilized buffer (20 mM Tris, 140 mM NaCl, 2 mM CaCl_2_ pH 8.0) and eluted overnight with gentle mixing in 25 ml of 0.5 M NaCl. The beads were then removed by centrifugation at 300×g for 5 minutes. The 0.5 M NaCl solution containing the eluted albumin was then buffer exchanged three times through a 10 kDa filter (EMD Millipore, Billerica, MD) using 15 ml Earle’s balanced salt solution (EBSS buffer) (Sigma, St. Louis, MO), tested for concentration using by absorbance at 280 nm, and diluted to a final concentration of 25 mg/ml in EBSS and stored at 4°C. Samples were diluted 1∶10 in 20 mM sodium phosphate buffer, pH 7.2, and run on 4–20% SDS gels (Bio-Rad, Hercules, California), which were silver stained to check for albumin purity.

### Depletion of Albumin

Albumin was depleted from human serum by affi-gel bead affinity elution (Bio-Rad). 500 µl of beads were washed three times in 2 ml PBS, and were added to 2 ml of serum with gentle mixing at room temperature for 2 hours. The beads were removed by centrifugation at 300×g for 5 minutes and the albumin depletion was repeated as above twice more. Samples were diluted 1∶10 in 20 mM phosphate buffer and run on 4–20% SDS gels which were silver stained to show differences in protein concentrations. Serum and depleted serum were diluted to 1∶100 and 1∶10 concentrations, respectively, and western blots were stained with mouse monoclonal anti human-albumin antibody, clone HSA-11, following the manufacturer’s instructions (Sigma).

### Trypsin Digest Products Added to Culture

TPCK-treated trypsin-coated agarose beads (Sigma) were washed according to the manufacturer’s instructions. To digest serum or albumin, 12.5 µl of beads was mixed with 250 µl of serum, 25 mg/ml albumin, 250 µl SFM containing 500 µg/ml human albumin or bovine albumin, or 250 µl serum-free medium containing 10 µg/ml insulin or 5 µg/ml transferrin. Beads were incubated with gentle rotation at 37 °C for 2 hours, and were removed by centrifugation at 300×g for 5 minutes. The digested media and undigested controls were mixed with PFM or SFM supplemented with bovine albumin. In experiments where human albumin was digested, the trypsinized human albumin and untrypsinized human albumin controls were, if indicated, mixed with SFM supplemented with human albumin.

### Collagen Staining by Flow Cytometry

24-well tissue culture treated plates (BD Bioscience, San Jose, CA) were coated for 1 hour at 37°C with 20 µg/ml human cellular fibronectin from fibroblasts (Sigma) in PBS and gently rinsed twice with sterile PBS. 500 µl of PBMC at 1×10^6^ cells/ml in SFM was added to each well. Control wells were supplemented with 250 µl SFM containing 500 µg/ml human albumin, while sample wells were supplemented with SFM digested by TPCK-treated trypsin-coated agarose beads as described above. After 5 days, cells were washed with warm PBS and exposed to 125 µl accutase (Innovative Cell Technologies, San Diego, CA) for 20 minutes at 37°C. Cells were resuspended via gentle pipetting, and washed by suspension in 1 ml ice cold PBS, collected by centrifugation at 300×g for 5 minutes, then washed once more. Cells were resuspended in 1% paraformaldehyde/0.2% saponin/PBS for 15 minutes on ice, washed twice as above, and resuspended in 2% BSA/0.2% saponin/PBS for 15 minutes of blocking. Anti-collagen type I rabbit (Rockland, Gilbertsville, PA) and control rabbit IgG (Jackson Immunoresearch, West Grove, PA) antibodies were added to the cell suspensions at 1 µg/ml and incubated on ice for 30 minutes. Cells were washed twice with ice cold PBS and resuspended in 4 µg/ml goat anti-rabbit alexa-fluor 488 secondary antibody (Molecular Probes, Eugene, OR) for 30 minutes on ice. Cells were washed twice and resuspended in ice cold PBS and analyzed with an Accuri C6 flow cytometer for fluorescence. Negative controls were used to set gates.

### Statistical Analysis

Statistics were performed using Prism (Graphpad software, San Diego, CA). Differences among multiple groups were assessed by 1-way ANOVA (with Dunnett’s post test), and between two groups by a two-tailed Mann-Whitney t-test. Significance was defined by p<0.05.

## Results

### Trypsin Treatment Increases Fibrocyte Number

**Figure 1:**
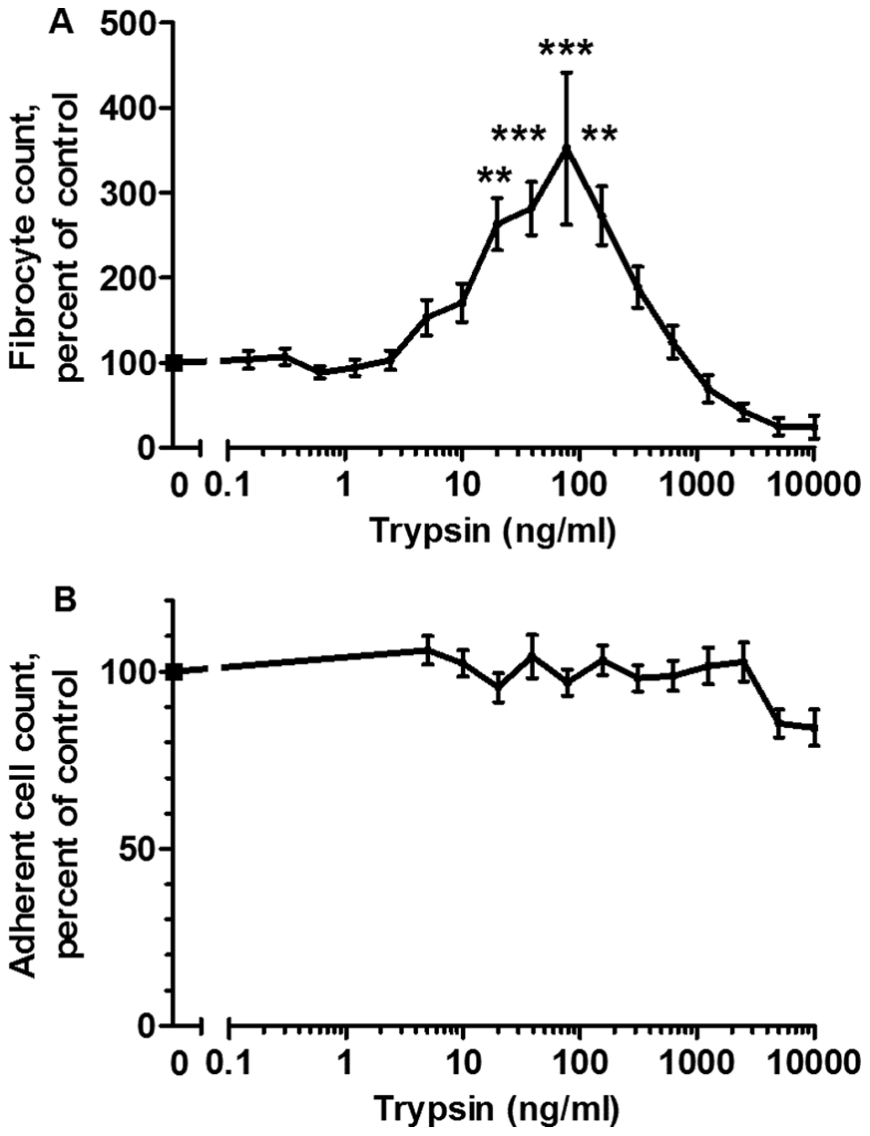
Trypsin potentiates fibrocyte differentiation. (**A**) PBMC were cultured in serum free media in the presence of the indicated concentrations of trypsin for 5 days, after which the PBMC were air-dried, stained, counted for fibrocyte differentiation, and normalized for each donor to the no-trypsin control. The no-trpysin controls developed 41.8±5.4 fibrocytes per 10^5^ PBMC. (**B**) The same PBMC populations were then counted for the total number of PBMC adhered to the plate following fixing and staining and normalized for each donor to the no-trypsin control. There were no significant differences in the numbers of adhered PBMC following fixing and staining. Values in A and B are mean ± SEM, n = 9. **indicates p<.01 and ***indicates p<.001 compared to the no-trypsin control by 1-way ANOVA, Dunnett’s test.

Topical treatment with trypsin improves wound healing, although the mechanism is unknown [48]–[54]. The differentiation of monocytes into fibrocytes plays a role in wound healing [10]–[12], [14]. To test the hypothesis that trypsin increases wound healing by potentiating fibrocyte differentiation, we examined the effect of trypsin on fibrocyte differentiation in culture. Human peripheral blood mononuclear cells (PBMC) were incubated with trypsin for 5 days in a defined serum-free medium. The cells were then stained and scored for fibrocyte formation. Fibrocyte numbers were normalized to trypsin-free controls due to variability in donors as we previously observed [17]–[20], [56]. Trypsin concentrations between 20 and 150 ng/ml significantly increased the number of fibrocytes (Figure 1A), and this effect was observed for all donors tested. Trypsin concentrations above 1000 ng/ml decreased the number of fibrocytes. The number of adherent cells following fixing and staining was not significantly affected by trypsin (Figure 1B), suggesting that trypsin specifically increases the number of differentiated fibrocytes, rather than increasing the general cell viability or adhesion. This suggests that trypsin can potentiate fibrocyte differentiation.

### Other Proteases do not Increase Fibrocyte Number

**Figure 2:**
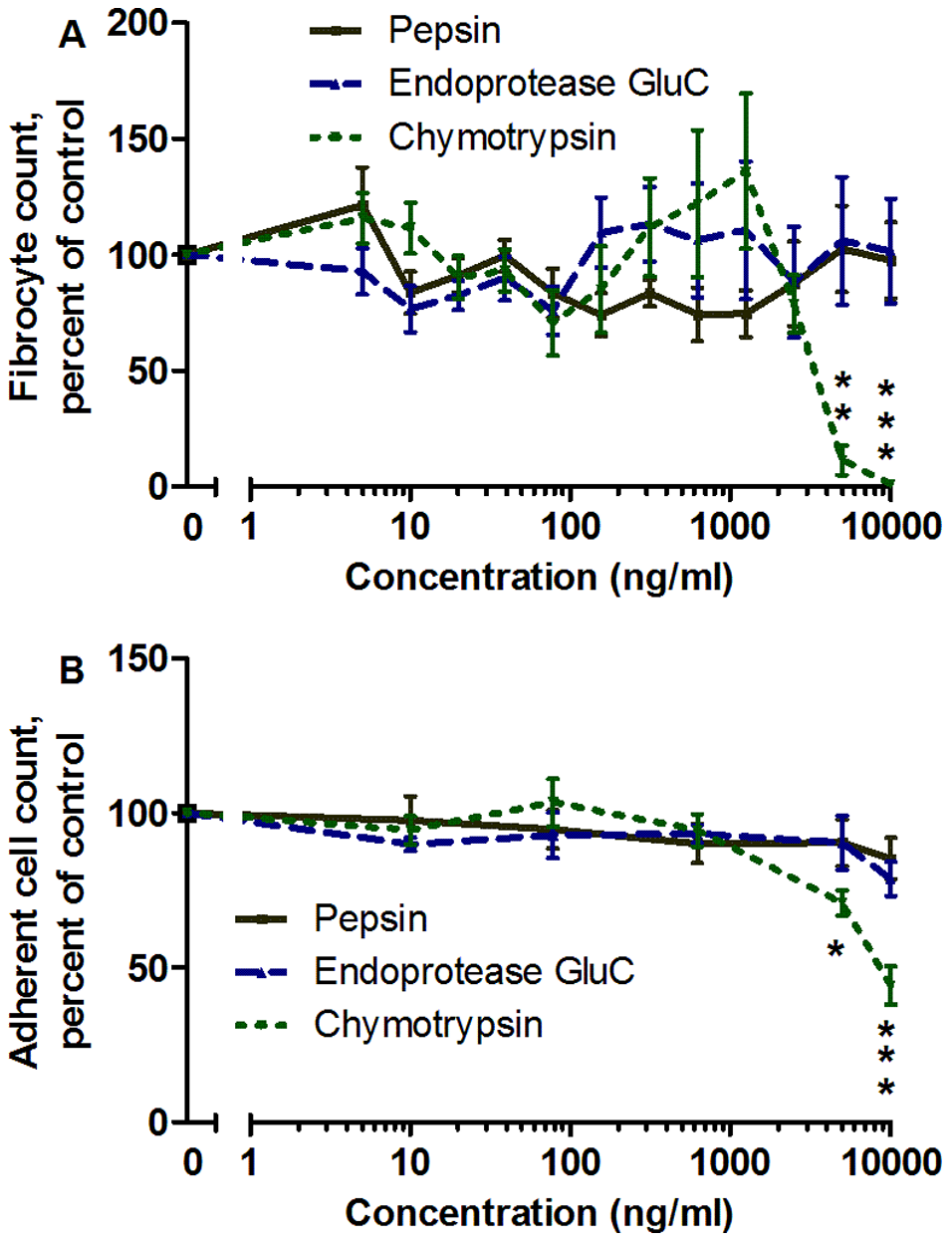
Chymotrypsin, pepsin, and endoproteinase GluC do not potentiate fibrocyte differentiation. (**A**) PBMC were cultured with the indicated concentrations of protease for 5 days, and fibrocytes were counted as in figure 1. (**B**) The same PBMC populations were then counted for the total number of PBMC adhered to the plate following fixing and staining, and normalized to the no-protease control. At high concentrations, chymotrypsin significantly lowered the numbers of fibrocytes and adhered PBMC. Values are mean ± SEM, n = 9 for pepsin and endoproteinase GluC, and n = 7 for chymotrypsin. *indicates p<.05, **indicates p<.01, and ***indicates p<.001 compared to the no-protease control by 1-way ANOVA, Dunnett’s test.

To determine if other proteases also potentiate fibrocyte differentiation, we examined the effect of three other proteases. Pepsin and endoproteinase Glu-C had no significant effect on fibrocyte differentiation or the number of adhered PBMC (Figure 2A and B). Chymotrypsin at 5000 ng/ml and above caused lower fibrocyte numbers and lower numbers of adhered PBMC (Figure 2A and 2B). These results suggest that not all proteases potentiate fibrocyte differentiation, and that a specific aspect of the protein structure or activity of trypsin is responsible for trypsin potentiating fibrocyte formation.

### Trypsin’s Enzymatic Activity causes Fibrocyte Potentiation

**Figure 3:**
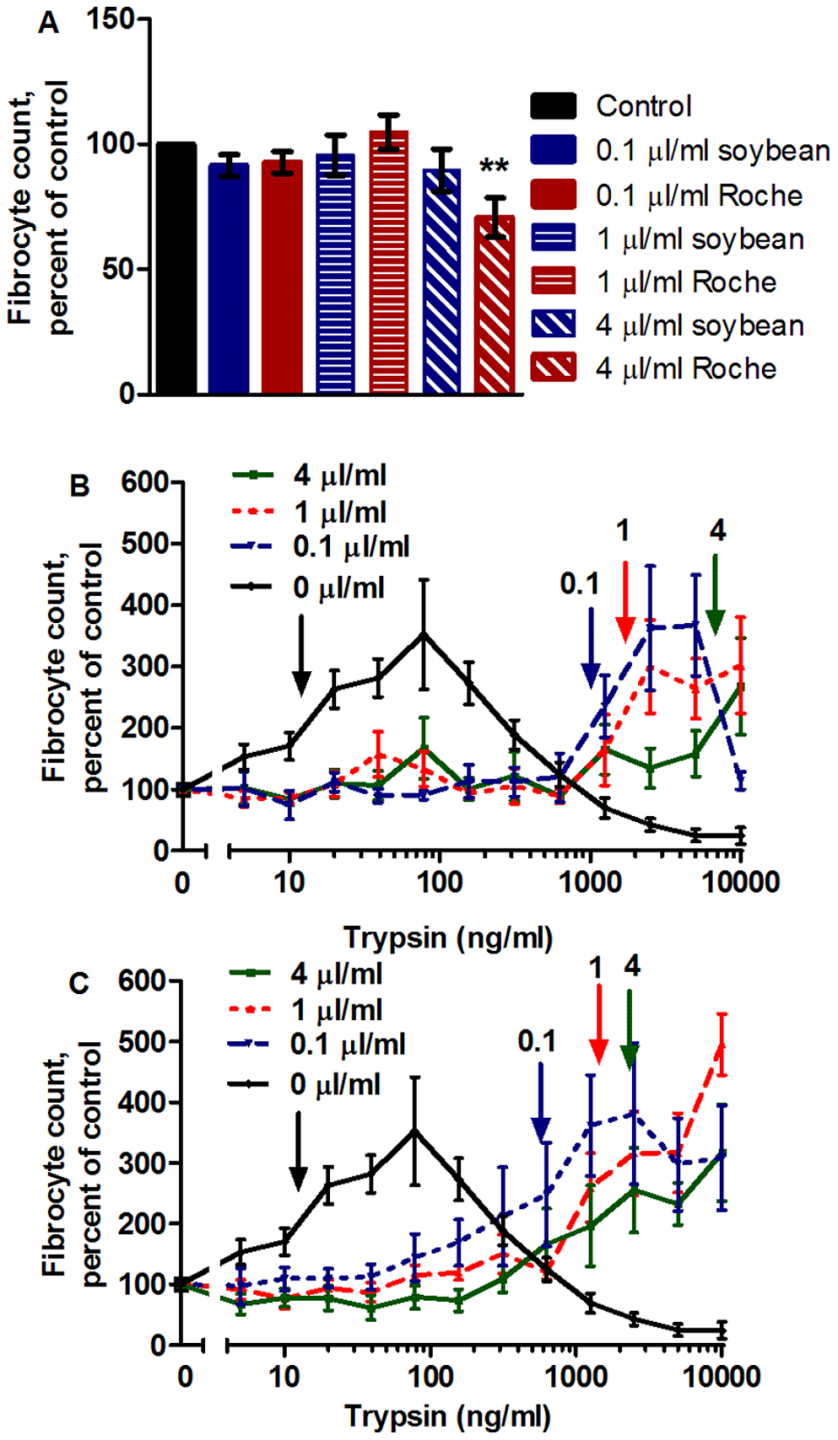
Trypsin inhibitors increase the amount of trypsin needed to potentiate fibrocyte potentiation. Trypsin inhibitors were added to PBMC cultures at the beginning of the 5-day incubation period at the indicated concentrations, with and without trypsin present. PBMC were then air dried, stained, counted for fibrocyte differentiation, and normalized to the no-trypsin control. (**A**) Trypsin inhibitors did not significantly affect fibrocyte differentiation with the exception of 4 µl/ml Roche inhibitor, which decreased fibrocyte formation. **indicates p<.01 compared to the inhibitor-free control. (**B**) Soybean trypsin inhibitor inhibited trypsin-induced fibrocyte potentiation. No-inhibitor data is the same as figure 1A. Fibrocyte counts were normalized to the inhibitor-containing trypsin-free control. Arrows indicate the lowest trypsin concentrations that doubled the fibrocyte number. Compared to the no-trypsin control, there was a significant increase in the number of fibrocytes with p<.05 for 4 µl/ml inhibitor for 10,000 ng/ml and at 1 µl/ml inhibitor for 5,000 ng/ml, and p<.001 at 1 µl/ml inhibitor for 2,500 and 10,000 ng/ml and at 0.1 µl/ml inhibitor for 2500 and 5,000 ng/ml (1-way ANOVA, Dunnett’s test). (**C**) Roche complete protease inhibitor cocktail inhibitor in the cell culture medium also inhibited trypsin-induced fibrocyte potentiation. No-inhibitor data is the same as figure 1A. Arrows indicate the lowest trypsin concentrations that doubled the fibrocyte number. Compared to the no-trypsin control, there was a significant increase in the number of fibrocytes with p<.05 at 4 µl/ml inhibitor for 1250 ng/ml and 0.1 µl/ml inhibitor for 1250 and 2500 ng/ml, p<.01 at 1 µl/ml inhibitor for 10,000 ng/ml and at 0.1 µl/ml inhibitor for 10,000 ng/ml, and p<.001 at 4 µl/ml inhibitor for 2500, 5000, and 10,000 ng/ml (1-way ANOVA, Dunnett’s test). Values are mean ± SEM, n = 7.

To determine whether trypsin’s enzymatic activity is necessary to potentiate fibrocyte formation, we examined the effect of two trypsin inhibitors on the ability of trypsin to potentiate fibrocyte differentiation. Adding trypsin inhibitors alone to cultures of PBMC had no significant effect on fibrocyte differentiation, with the exception of 4 µl/ml protease inhibitor cocktail, which decreased overall fibrocyte formation (Figure 3A). The addition of soybean trypsin inhibitor (Figure 3B) or protease inhibitor cocktail (Figure 3C) increased the amount of trypsin needed to potentiate fibrocyte differentiation. Increasing the inhibitor concentration increased the concentration of trypsin necessary to double fibrocyte differentiation compared to the controls (Arrows, Figures 3B and 3C). Pre-incubating trypsin and inhibitor completely abrogated trypsin’s potentiation of fibrocyte differentiation (data not shown). Together, these results suggest that the protease activity of trypsin affects its ability to potentiate fibrocyte differentiation.

### Albumin is Necessary for Trypsin to Potentiate Fibrocyte Differentiation

**Figure 4:**
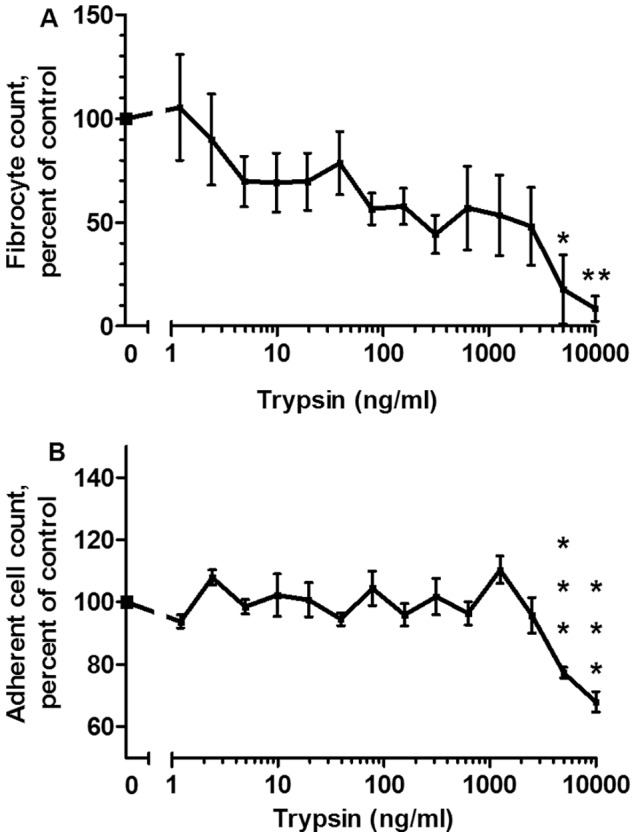
Trypsin does not potentiate fibrocyte differentiation in medium lacking protein supplements. (**A**) PBMC were cultured with the indicated concentrations of trypsin for 5 days in protein-free medium, after which the PBMC were air-dried, stained, counted for fibrocyte differentiation, and normalized to the no-trypsin control. The no-trypsin controls developed 39±7.2 fibrocytes per 10^5^ PBMC. (**B**) The same PBMC populations were then counted for the total number of PBMC adhered to the plate following fixing and staining. Values are mean ± SEM, n = 7. *indicates p<.05, **indicates p<.01, and ***indicates p<.001 compared to the no-trypsin control by 1-way ANOVA, Dunnett’s test.

To test the hypothesis that trypsin acts on a protein supplement in the media to potentiate fibrocyte differentiation, we removed the protein supplements from our defined medium and added trypsin to this protein-free media. According to the manufacturer, Fibrolife medium is protein-free. Trypsin added to Fibrolife media lacking all three protein supplements (albumin, insulin and transferrin) did not potentiate fibrocyte differentiation (Figure 4A). At concentrations of 5 µg/ml and higher, trypsin significantly decreased both fibrocyte numbers and the number of adhered cells (Figures 4A and 4B), presumably by decreasing cell adhesion. These results suggest that trypsin acts on a protein supplement to indirectly potentiate fibrocyte differentiation, or that a protein supplement is necessary for trypsin’s potentiation to occur.

**Figure 5:**
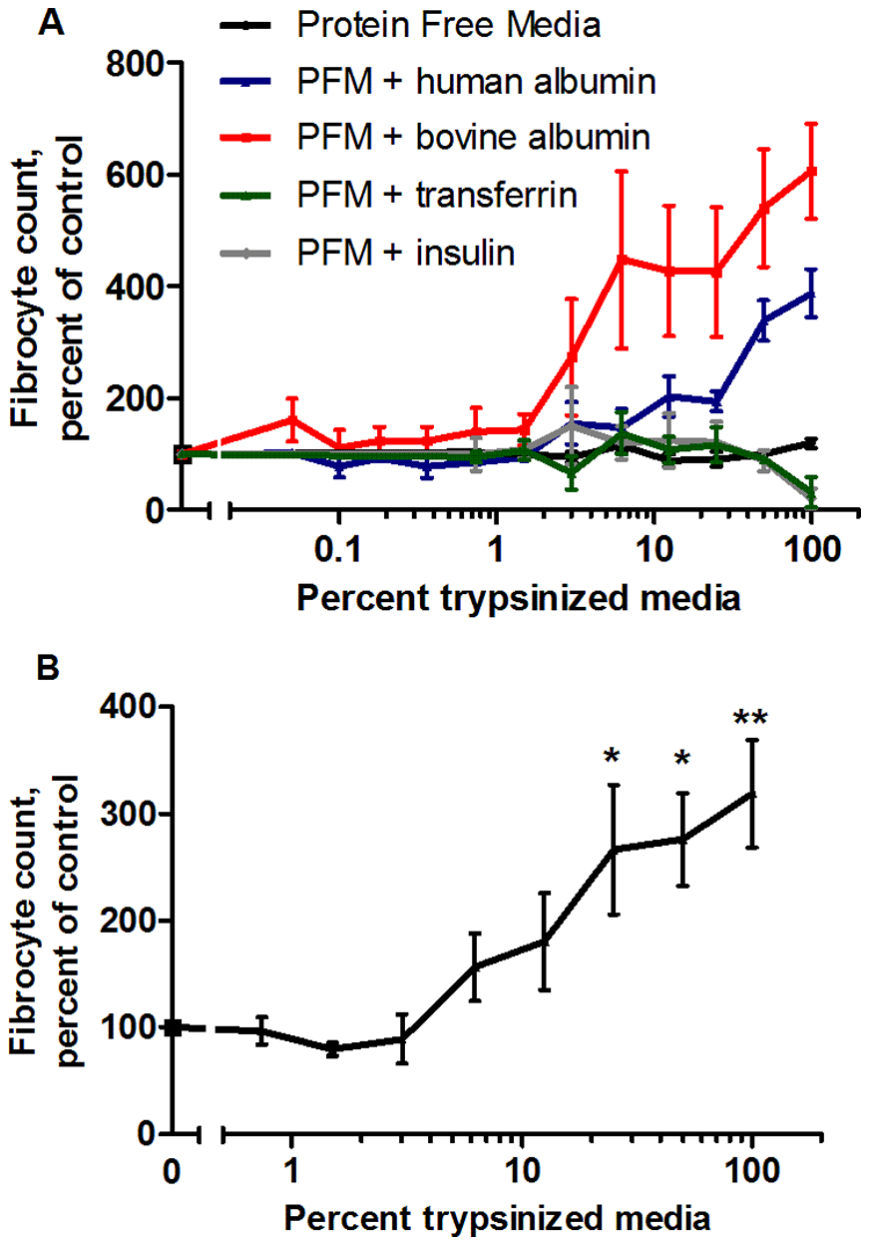
Serum-free medium containing albumin potentiates fibrocyte differentiation after temporary mixing with trypsin-coated agarose beads. TPCK-treated trypsin-coated agarose beads were used to digest protein-free culture media (PFM) and PFM containing bovine albumin, human albumin, transferrin, or insulin, after which the beads were removed. Digested media and controls were mixed with SFM at the indicated percentages and added to PBMC at the beginning of a 5 day incubation, after which the PBMC were air-dried, stained, counted for fibrocyte differentiation, and normalized to the control media containing the same amount of undigested protein. (**A**) After removal of the beads, only medium containing albumin fragments potentiated fibrocyte differentiation. Values are mean ± SEM, n = 3 for protein-free medium (PFM), insulin, and transferrin, n = 4 for bovine albumin, and n = 7 for human albumin. Compared to the human albumin control, digested human albumin significantly increased the number of fibrocytes with p<.05 at 62 µg/ml, p<.01 at 250 µg/ml, and p<.001 at 500 µg/ml. Compared to the bovine albumin control, digested bovine albumin increased the number of fibrocytes with p<.05 at 31 µg/ml, p<.01 at 250 µg/ml, and p<.001 at 500 µg/ml (1-way ANOVA, Dunnett’s test). (**B**) Human albumin-containing media potentiated fibrocyte differentiation when digested by non-TPCK treated trypsin-coated agarose beads. Values are mean ± SEM, n = 3. *indicates p<.05 and **indicates p<.01 compared to the human albumin control by 1-way ANOVA, Dunnett’s test.

**Figure 6:**
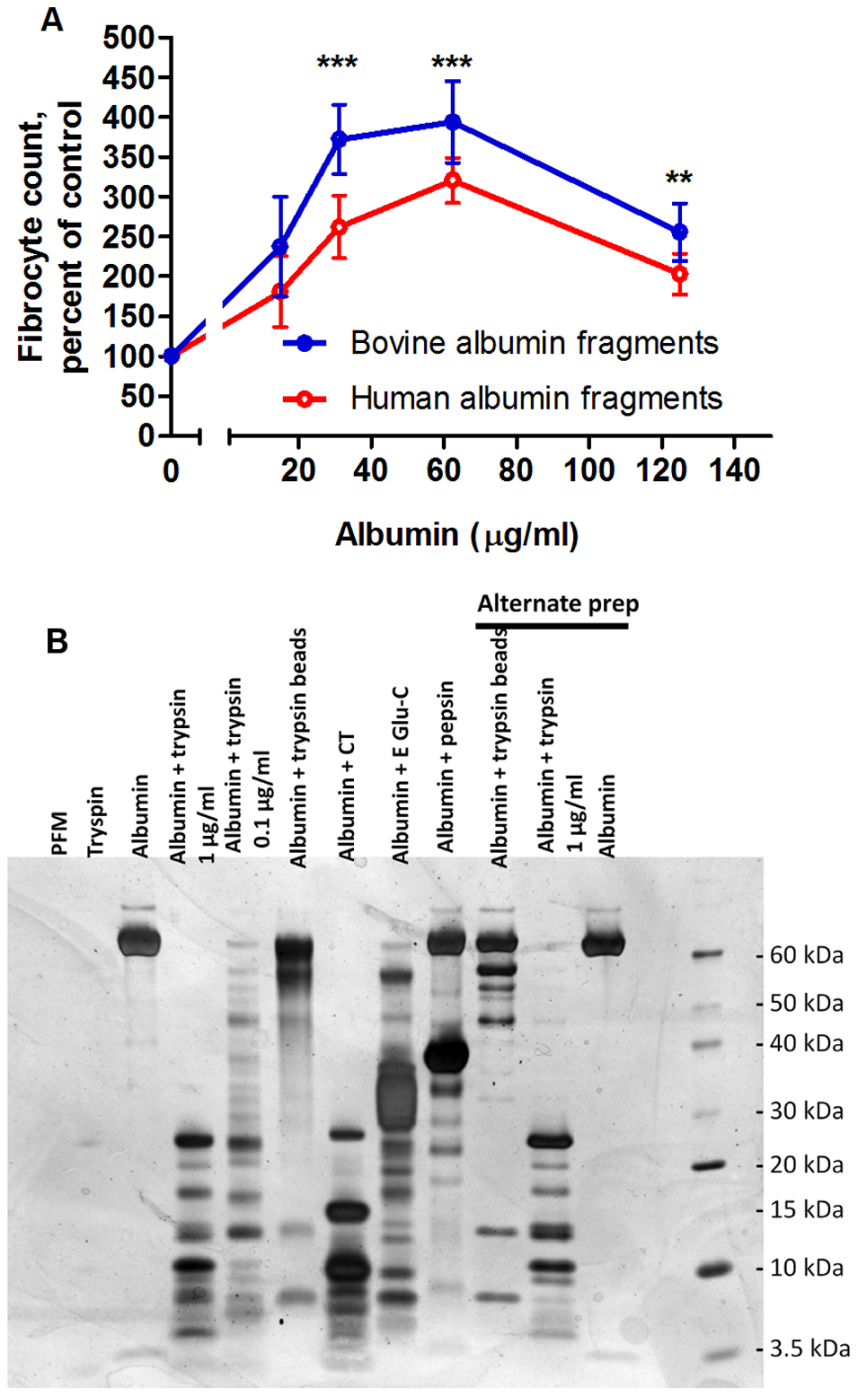
Albumin potentiates fibrocyte differentiation after temporary mixing with trypsin-coated agarose beads. TPCK-treated trypsin**-**coated agarose beads were used to digest bovine or human albumin. After the beads were removed, the digested albumin was added to PBMC cultures in SFM at the indicated concentrations for 5 days, after which the PBMC were air-dried, stained, counted for fibrocyte differentiation, and normalized to the bovine or human albumin controls. Values are mean ± SEM, n = 4, **indicates p<.01 and ***indicates p<.001 for both bovine and human albumin compared to the no-albumin control (1-way ANOVA, Dunnett’s test). (**B**) 250 µl of protein-free media supplemented with human albumin was digested for two hours with gentle rotation at 37°C with 1 µg/ml protease, 0.1 µg/ml protease, or with 12.5 µg/ml TPCK-treated trypsin-coated agarose beads. Samples were then run on a 7.5% SDS-PAGE gel and silver stained. PFM indicates protein free medium, CT indicates chymtotrypsin, and Glu-C indicates endoprotease Glu-C. Alternative prep indicates samples from a repeat of this experiment that happened to be included in this gel. This gel is a digestion of human albumin, and is representative of similar gels of digestions of medium containing bovine albumin or ITS-3.

Serum-free media contains three proteins: insulin, transferrin, and albumin. To determine whether insulin, transferrin or albumin potentiates fibrocyte differentiation when exposed to trypsin, we purified human and bovine albumin and made media containing only insulin, transferrin, or albumin. When TPCK-treated trypsin-coated agarose beads were used to trypsinize culture media containing purified human or bovine albumin, the trypsinized media potentiated fibrocyte formation following the removal of the beads and addition to PBMC (Figure 5A). Fibrocyte potentiation did not occur after the addition of protein-free, insulin-containing, or transferrin-containing media trypsinized in the same fashion (Figure 5A). To verify that TPCK did not influence fibrocyte differentiation, we digested human-albumin containing SFM with non TPCK-treated trypsin beads. Media containing human albumin digested by non TPCK-treated trypsin-beads also potentiated fibrocyte differentiation (Figure 5B). Using trypsin-coated beads to directly digest bovine and human albumin into fragments, and then adding those fragments to protein-free medium also potentiated fibrocyte differentiation compared to undigested controls (Figure 6A). SDS-PAGE gels indicated that the protease treatment of albumin caused the formation of digestion products (Figure 6B). These results suggest that a trypsin fragment of albumin may potentiate fibrocyte differentiation.

### Increased Fibrocyte Numbers are Associated with Increased Collagen Expression

**Figure 7:**
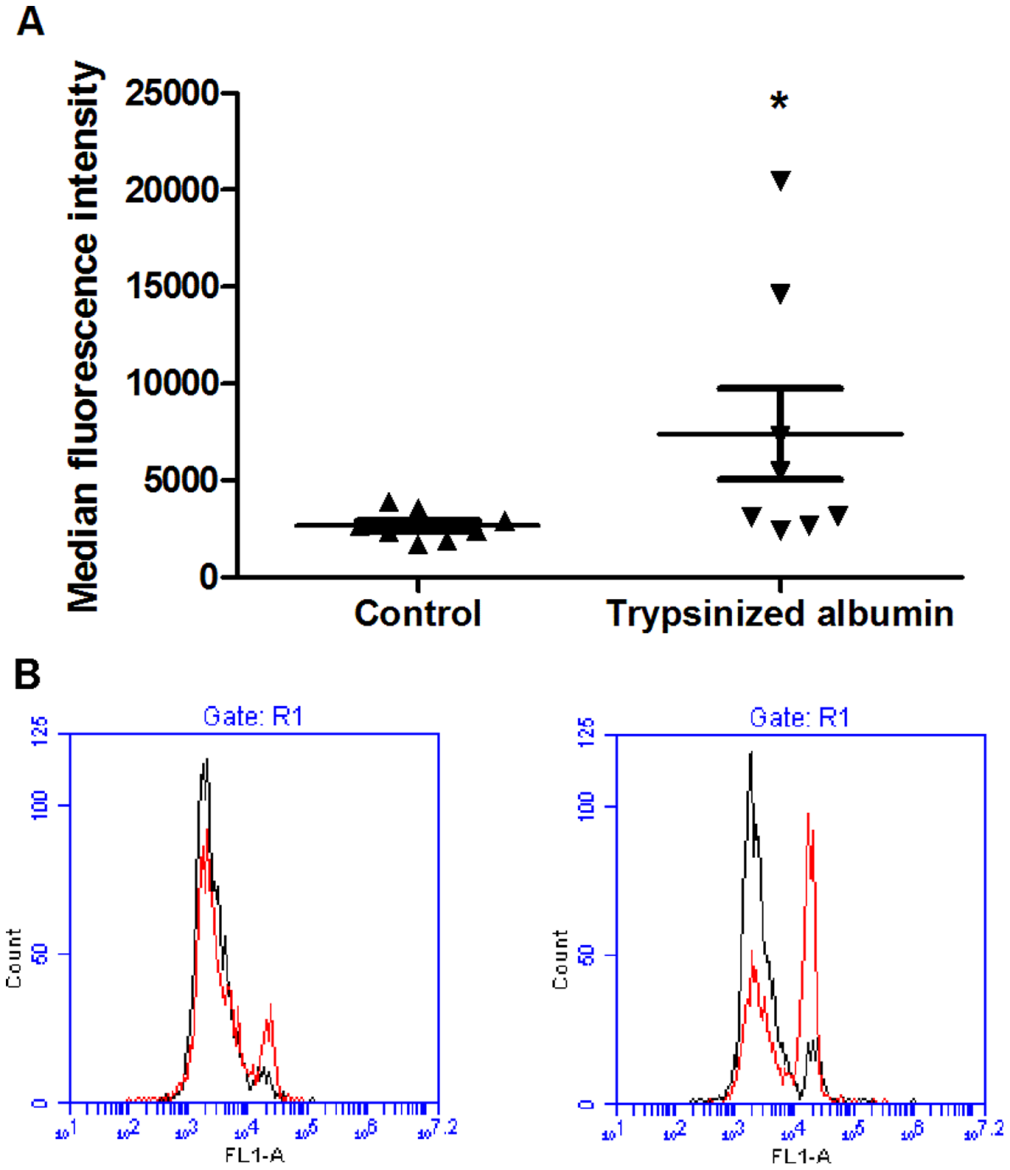
Collagen production is increased in PBMC exposed to trypsinized albumin. (**A**) TPCK-treated trypsin**-**coated agarose beads were used to digest protein-free media and media containing human albumin. After the beads were removed, the media was added to PBMC cultures in serum-free medium at a 33% concentration. Cells were exposed to trypsinized human albumin for 5 days, then resuspended, stained with anti-collagen and alexa-fluor secondary antibodies, and measured for fluorescence by flow cytometry. Values are mean ± SEM, n = 9, *indicates p<.05 by two tailed Mann- Whitney’s t-test. (**B**) Representative fluorescence overlay for collagen staining. The cells in the left plot were stained with isotype control primary antibodies, while the cells in the right plot were stained with anti-collagen primary antibodies. Red indicates trypsin-treated cells, while black indicates no trypsin treatment of the cells.

Increased collagen expression in a PBMC culture is a marker for increased fibrocyte differentiation [10], [11], [55]. To determine whether trypsinized human albumin increases collagen-positive cells, we resuspended and stained PBMC with an anti-collagen antibody following our 5-day differentiation assay. Increased collagen expression was detected by flow cytometry in PBMC cultures incubated with trypsinized human albumin (Figures 7A). A representative flow plot can be seen in Figure 7B. This suggests that the albumin fragment-induced increase in fibrocyte number is accompanied by an increase in collagen expression.

### Trypsinized Albumin Increases Fibrocyte Formation in an Enriched Monocyte Population

Fibrocytes differentiate from monocytes [10], [12], [17]. Cells in a PBMC population can include T-cells, B-cells, or NK cells [20]. To determine whether trysinized albumin acts directly on monocytes to potentiate fibrocyte differentiation, as opposed to an indirect action through other cells in the PBMC population, we isolated monocytes by negative selection from an average of 16% to an average of 83% purity. When added to the monocyte-enriched cells, 70 µg/ml trypsinized albumin potentiated fibrocyte differentiation by 223% ±9% (n = 3; p<0.01, t-test). This suggests that tryptic fragments of albumin act directly on monocytes to potentiate fibrocyte differentiation.

### Trypsinizing Albumin-containing Serum Promotes Fibrocyte Differentiation

**Figure 8:**
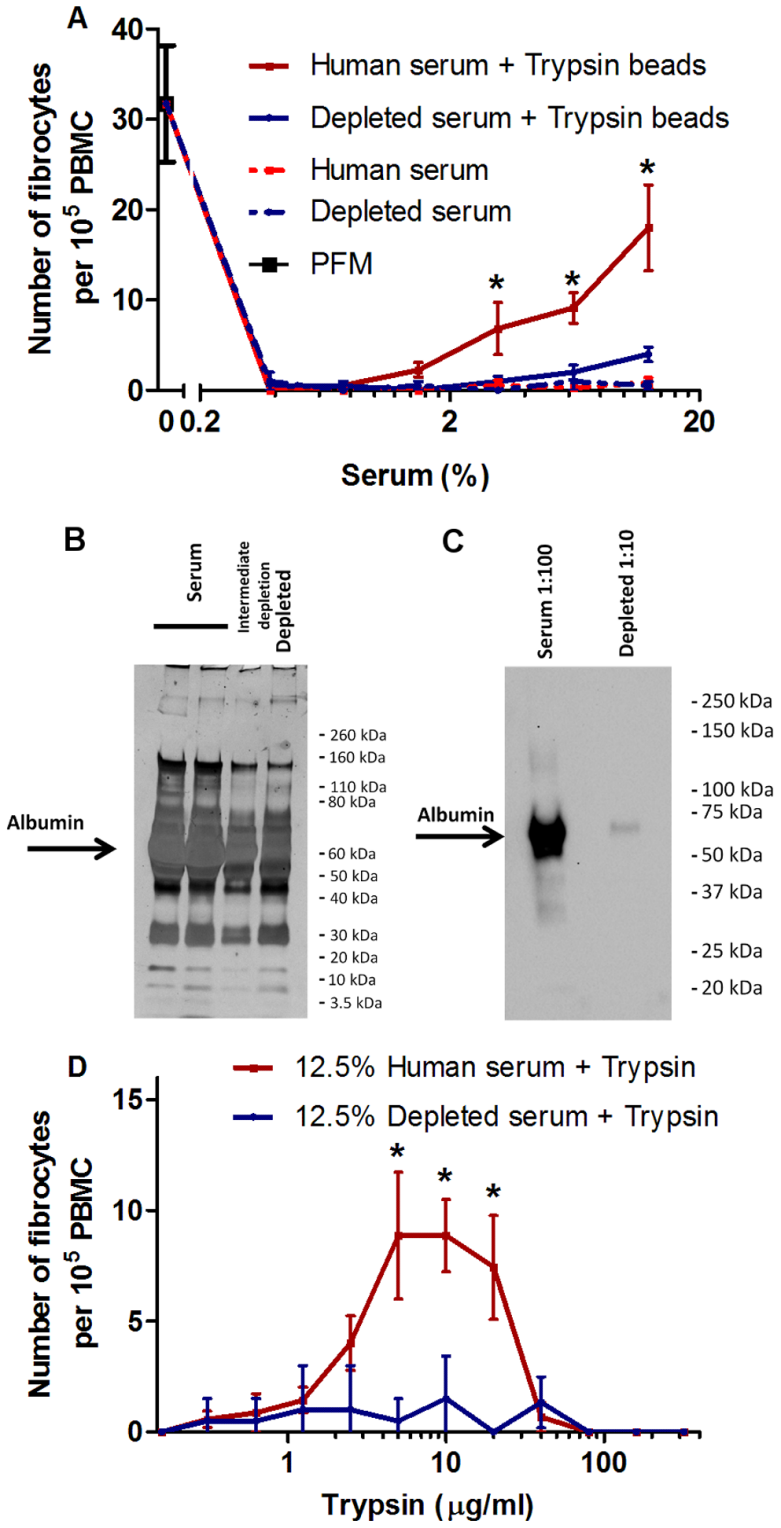
Trypsinized human serum containing albumin potentiates fibrocyte differentiation. (**A**) TPCK-treated trypsin-coated agarose beads were used to digest human serum and albumin-depleted human serum, which were then added to PBMC in protein-free media at the indicated concentrations for 5 days, after which the cells were air-dried, stained, counted for fibrocyte differentiation. Values are mean ± SEM, n = 7 for serum, n = 4 for depleted serum. Compared to the trypsinized depleted serum, trypsinized serum significantly increased the number of fibrocytes: *indicates p<.05 by a two-tailed Mann-Whitney’s t-test. Compared to depleted serum, trypsinized depleted serum did not significantly increase fibrocyte number by a two-tailed Mann-Whitney’s t-test. (**B**) Albumin-depleted serum shows less albumin, but a similar protein pattern to human serum, when both are run at 1∶10 dilutions on a silver stained 4–20% SDS-PAGE gel. (**C**) A Western blot of serum and albumin-depleted serum stained with anti human-albumin antibodies. (**D**) Trypsin was added to PBMC in a 12.5% serum containing medium, and a 12.5% albumin-depleted serum containing medium, at the indicated concentrations for 5 days, after which the cells were air-dried, stained, counted for fibrocyte differentiation. Values are mean ± SEM, n = 7 for serum, n = 4 for depleted serum. Compared to the trypsinized depleted serum, trypsinized serum significantly increased the number of fibrocytes: *indicates p<.05 by a two-tailed Mann-Whitney’s t-test.

In a wound environment, monocytes and exogenous trypsin would be exposed to serum. Human serum contains more than 500–1200 proteins, of which the primary component is albumin [57], [58]. To determine whether trypsin potentiates fibrocyte differentiation when mixed with human serum, we digested human serum with trypsin, and then added these digestion products to PBMCs. As previously observed [20], human serum inhibited fibrocyte differentiation, presumably due to the presence of serum amyloid P (SAP) in the serum (Figure 8A). Compared to no trypsin, trypsin-treated serum increased fibrocyte differentiation. When the serum was depleted of albumin (Figures 8B and 8C), the serum also inhibited fibrocyte differentiation, and compared to no trypsin, trypsin treatment did not cause an increase in the number of fibrocytes (Figure 8A). Compared to the no-trypsin control, in media containing 12.5% human serum, trypsin concentrations between 5 and 20 µg/ml increased fibrocyte differentiation, while the same concentrations of trypsin in albumin-depleted serum media did not increase fibrocyte differentiation (Figure 8D). These results indicate that trypsin potentiation of fibrocyte differentiation in serum requires albumin.

## Discussion

Trypsin speeds the healing of dermal wounds [48]–[54]. Albumin is a major component of serum, and a trypsin-treated extract of serum potentiates wound healing [49]. In this report, we showed that trypsin potentiates monocyte differentiation into fibrocytes in culture and that albumin is necessary for this potentiation to occur. This then suggests that topical trypsin and trypsin-treated serum potentiates wound healing by tryptic fragments of albumin potentiating fibrocyte differentiation.

Trypsin inhibitors in the culture medium of PBMC caused more trypsin to be necessary to potentiate fibrocyte differentiation, indicating that trypsin’s enzymatic activity is the key factor in albumin digestion and fibrocyte potentiation. Soybean trypsin inhibitor is a slow-binding but nearly irreversible inhibitor of trypsin’s enzymatic activity [59]. Soybean trypsin inhibitor stoichiometrically binds trypsin, and the amount of soybean-trypsin inhibitor added to the trypsin-containing PBMC cultures at higher concentrations was in excess of the total amount of trypsin added. Human serum also contains reversible protease inhibitors with less binding efficacy for trypsin than soybean inhibitor [60], [61]. When mixed and immediately added to albumin-containing medium, not even the highest concentration of soybean inhibitor or serum (Figures 3B and 8D) completely abrogated trypsin’s albumin-induced fibrocyte potentiation, suggesting that transient trypsinization is enough to digest albumin and induce fibrocyte differentiation.

Increased fibrocyte formation correlates with increased fibrosis [62] and faster wound healing [16], and protein additives to wound dressings can improve the wound healing response [16]. Chronic non-healing wounds are often resistant to more usual treatment dressings [6], [7]. Chronic wounds are associated not only with infection, age, and diabetes, but also with decreased albumin concentrations in the wound area [24]–[27]. An intriguing possibility is that if albumin degradation products in wounds potentiate fibrocyte differentiation, the decreased albumin concentrations in the chronic wounds might result in lower levels of the albumin degradation products in the chronic wounds, resulting in lower levels of fibrocyte differentiation.

Trypsin at ∼50 mg/L (50 µg/ml) has been used to produce a lyophilized, trypsinized serum for wound treatment [49]. We observed that 5–20 µg/ml trypsin added to 12.5% serum potentiates fibrocyte differentiation (Figure 8D). This would then correspond to 40–160 µg/ml trypsin in 100% serum, which corresponds to the trypsin concentration used for the wound-healing product.

Proteinases have previously been implicated in interactions with proteinase-activated receptors (PARs) [45]. PARs are activated by cleavage of a small peptide from the surface of the receptor by a serine protease, usually trypsin or chymotrypsin [45]. Research on PARs has been primarily confined to mesenchymal cells, usually in the digestive tract where trypsin is a common enzyme [63]–[66]. Since cells treated with trypsin in protein-free media do not have increased fibrocyte formation, and chymotrypsin does not potentiate fibrocyte differentiation, trypsin’s effect on fibrocyte differentiation does not appear to be mediated by proteinase activated receptors.

Monocyte-derived fibrocytes are found in wound healing environments [10]–[12] and at tissue near a tumor edge [67]–[69], both areas of increased serine proteinase activity [38], [70]–[73]. For instance, marapsin, a protease that also cleaves at arginine, is up-regulated in wound healing environments [70]. An intriguing possibility is that endogenous proteases, by generating tryptic fragments of albumin, help to potentiate fibrocyte differentiation in wounds and tissues near a tumor edge.

Taken together, our results suggest that topical trypsin and trypsinized albumin potentiate wound healing at least in part by potentiating fibrocyte differentiation. While trypsin has been used in the treatment of burns and in wound dressings for more than 50 years, a mixture of albumin and trypsin may further speed wound healing, especially when applied to chronic wounds deficient in albumin [24]–[27].
